# Clinical Effects of Asynchronous Provider-Guided Practice Sessions During Blended Care Therapy for Anxiety and Depression: Pragmatic Retrospective Cohort Study

**DOI:** 10.2196/60502

**Published:** 2024-10-18

**Authors:** Hallie M Espel-Huynh, Lu Wang, Emily G Lattie, Robert E Wickham, Alethea Varra, Connie E Chen, Anita Lungu, Jennifer L Lee

**Affiliations:** 1 Lyra Health Burlingame, CA United States; 2 Department of Psychological Sciences Northern Arizona University Flagstaff, AZ United States

**Keywords:** anxiety, depression, blended care therapy, guided practice sessions, mental health care, digital mental health, psychotherapy outcomes

## Abstract

**Background:**

Blended care therapy models are intended to increase the efficiency and effectiveness of evidence-based psychotherapy by combining synchronous and asynchronous components of care.

**Objective:**

This retrospective cohort study evaluated the clinical effects of synchronous video therapy sessions and asynchronous guided practice session elements on anxiety and depression in a blended care therapy program, with a novel focus on asynchronous provider feedback messages.

**Methods:**

Participants were adults (N=33,492) with clinical symptoms of anxiety (Generalized Anxiety Disorder 7-item scale [GAD-7] score of ≥8) and depression (Patient Health Questionnaire 9-item scale [PHQ-9] score of ≥10) at intake. Symptom trajectories were evaluated via individual growth curve models. Time-varying covariates evaluated effects of synchronous video session attendance and the presence or absence of each asynchronous guided practice session element occurring within 7 days and 8-14 days prior to each clinical outcome assessment. Guided practice session elements included client digital lesson completion, client digital exercise completion, and feedback messages sent by providers.

**Results:**

Approximately 86.6% (29,012/33,492) of clients met criteria for clinical improvement by end of care (median 6, IQR 4-8 synchronous sessions). Synchronous video session attendance and client digital lesson completion in the past 7 days and in the past 8-14 days were each uniquely and significantly associated with lower GAD-7 scores (video session effects: *b*_session7_=–0.82, *b*_session8-14_=–0.58, *P* values<.001; digital lesson effects: *b*_lesson7_=–0.18, *b*_lesson8-14_=–0.26, *P* values <.001) and PHQ-9 scores (video session effects: *b*_session7_=–0.89, *b*_session8-14_=–0.67, *P* values <.001; digital lesson effects: *b*_lesson7_=–0.12, *b*_lesson8-14_=–0.30, *P* values <.001). Client digital exercise completion in the past 8-14 days was significantly associated with lower GAD-7 scores (*b*_exercise8-14_=–0.10; *P*<.001) but exercise completion in the 7 days prior to clinical outcome assessment was not (*b*_exercise7_=0.00; *P*=.89). Exercise completion in the past 7 days was significantly associated with lower PHQ-9 scores (*b*_exercise7_=–0.16; *P*<.001) but exercise completion in the past 8-14 days was not (*b*_exercise8-14_=–0.05; *P*=.09). Provider feedback messaging in the past 7 days and in the past 8-14 days was significantly associated with lower GAD-7 and PHQ-9 scores, respectively (GAD-7: *b*_feedback7_=–0.12, *P*<.001; *b*_feedback8-14_=–0.07, *P*=.004; PHQ-9: *b*_feedback7_=–0.15, *P*<.001; *b*_feedback8-14_=–0.08, *P*=.01).

**Conclusions:**

Provider feedback between synchronous therapy sessions provided significant benefit for symptom reduction, beyond the effects of client digital engagement and synchronous video sessions. When guided practice sessions are well integrated into care, blended care therapy provides meaningful improvements upon the traditional, synchronous session–only therapy model. Provider guidance and feedback for clients between synchronous sessions support more efficient and effective mental health care overall.

## Introduction

Evidence-based psychological treatments for anxiety and depression (eg, cognitive behavioral therapy) are highly effective in reducing symptoms and improving functioning [1-3]. These evidence-based psychotherapies include “active elements” such as therapy concepts, emotion regulation skills, and behavior change skills that impart unique therapeutic benefits [4,5]. Clients benefit from these active elements through a multistage process of provider sharing, client understanding, and client application of skills and concepts [5]. In traditional therapy models, therapists *share* or teach the active elements of these treatments solely during live, synchronous, once-weekly sessions. To fully benefit, clients must take in and *understand* this information as it pertains to their experience and then *apply* it independently via “homework” assignments that are completed between therapy sessions [5]. More consistent homework completion is associated with greater symptom reductions in psychotherapy [6,7]. However, multiple inefficiencies to this model have been identified. In the traditional model, clients often struggle to complete homework independently, with less than 40% of homework typically completed as assigned [8,9]. In addition, therapists follow up on homework with clients only approximately 50% of the time, which reduces opportunities to reinforce and consolidate client learning [10,11]. Without sufficient out-of-session skills practice, clients tend to experience suboptimal outcomes, including increased likelihood of premature treatment dropout, slower symptom improvements (presumably due to slower skills acquisition), and ultimately worse symptoms at end of care [7,12-14]. The traditional therapy model is therefore limited in the extent to which it supports client understanding, retention, and application of the skills and concepts that are necessary for treatment effectiveness [11].

Blended care therapy models have emerged as a potential solution to these challenges. These treatments bridge the gap between synchronous therapy sessions with asynchronous therapeutic engagement and digital content [15]. Preliminary evidence for blended care therapy models suggests that they can produce comparable or significantly better clinical outcomes in fewer live therapy sessions, relative to face-to-face psychotherapy [16,17]. Blended care therapy is an umbrella term that encompasses many treatment approaches. These approaches vary widely in the extent of asynchronous provider engagement between synchronous sessions, as well as the degree to which digital content is fully integrated into care [15]. This paper specifically examines a model of blended care therapy called “Lyra Care Therapy” (LCT). LCT blends synchronous video therapy sessions with intensive asynchronous *guided practice sessions*. In guided practice sessions, clients learn new evidence-based therapy concepts (beyond what is learned in synchronous sessions), receive messaging-based support from their providers, and apply therapy skills in their daily lives. Prior research on the LCT program has shown that this type of care is highly effective, with 89% of clients achieving either reliable improvement or recovery on validated outcome measures, and 74% achieving both reliable improvement and recovery [18]. This is substantially higher than the treatment response rates for depression found in standard, face-to-face mental health care settings. For example, a recent meta-analysis estimated that depression treatment response rates in “usual care” mental health treatment are approximately 20% [19].

LCT guided practice sessions contain multiple components, including digital video lessons, digital exercises, client message exchanges with their providers, and direct provider feedback to clients on their exercises. During synchronous sessions, providers set clients up for success by collaborating to select relevant and clinically appropriate digital activities to be completed. Clients then complete digital video lessons and exercises asynchronously during the guided practice session. Digital video lessons reinforce therapeutic skills taught in synchronous sessions or teach new concepts. Digital exercises then support clients in applying their newly learned skills in daily life, which is essential to facilitate symptom reductions [20].

Providers play a key role during LCT guided practice sessions by viewing clients’ progress on digital activities in the secure platform, sharing prompt feedback messages to reinforce clients’ efforts, and providing support through direct client-provider messaging. At their core, guided practice sessions offer a more engaging and effective alternative to traditional “homework” assignments in psychotherapy by facilitating asynchronous provider support and providing a more enriching digital experience for clients. Information from clients’ digital engagement also complements providers’ impressions from synchronous sessions to inform case conceptualization and care planning.

Past research evaluating this specific blended care model has demonstrated its effectiveness for reducing symptoms of anxiety and depression, including across diverse racial and ethnic groups and in large samples of up to approximately 6000 participants [18,21]. A prior LCT component analysis study also provided preliminary support for the role of client engagement with guided practice sessions in improving symptoms [22]. However, this prior component analysis focused exclusively on client engagement and did not assess the role of provider engagement. In LCT, the provider also plays a key role in supporting guided practice, by curating what is assigned, providing positive reinforcement, answering client questions, and offering corrective feedback when needed. Prior results from research on homework effectiveness in evidence-based psychotherapies suggest that these provider-level factors may also contribute to therapy effectiveness [11,23,24]. In traditional therapy models, however, provider support is limited to in-session interactions. In contrast, LCT providers additionally give feedback and encouragement to their clients between synchronous sessions via written messages in the platform. Thus, clients can readily access them and apply the feedback to their daily lives.

Although there is theoretical support for the importance of provider engagement in a blended care model, there is no known research evaluating the link between provider-specific digital engagement and clinical therapy outcomes. Therefore, a primary aim of this study was to explore the clinical impact of digital provider feedback in LCT, alongside client-focused engagement during guided practice sessions. We hypothesized that engagement with each element of the LCT program (ie, session attendance, client digital lesson completion, client digital exercise completion, and provider feedback) in the 7 days and 8-14 days prior to an outcome assessment would be associated with lower symptom severity at that assessment.

## Methods

### Study Design and Setting

This observational study used a retrospective cohort design and is reported in line with guidelines for STROBE (Strengthening the Reporting of Observational Studies in Epidemiology; see Multimedia Appendix 1 for STROBE checklist). Participants were adults in the United States who were eligible for an employer-sponsored mental health benefit, Lyra Health, offered by Lyra Clinical Associates. Individuals seeking care completed a brief, questionnaire-based assessment via a secure web-based platform to evaluate baseline symptoms and recommend appropriate care. All care activities were conducted via a proprietary, HIPAA (Health Insurance Portability and Accountability Act)–compliant platform that is accessible via web browser and mobile device.

### Ethical Considerations

This deidentified data analysis was determined to be exempt by the WCG institutional review board (WCG IRB Tracking ID#20220388). Participants provided informed consent to take part in care and have their deidentified data used for research purposes as a part of that consent for care. Data were collected as part of routine practice in the LCT program to support measurement-based clinical care and for quality assurance. Participants did not receive compensation for engagement with the LCT program or for completing assessments.

### Participants

**Figure 1:**
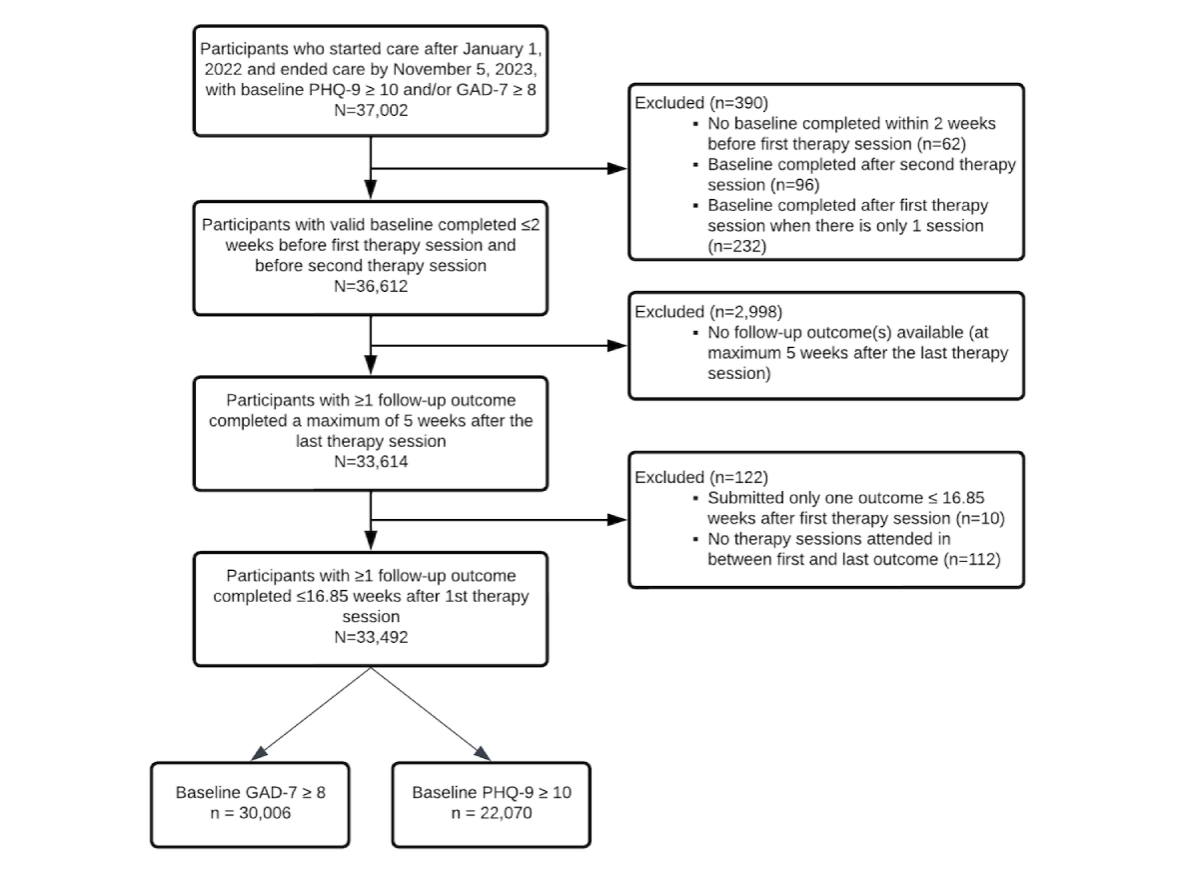
Participant inclusion and exclusion. GAD-7: Generalized Anxiety Disorder—7-item scale; PHQ-9: Patient Health Questionnaire-9 item scale.

Individuals were eligible for this study if they began care on or after January 1, 2022, and completed or dropped out of care by November 5, 2023. This time frame was selected to allow for accumulation of a large sample size over nearly 2 years that was sufficiently representative and diverse to provide adequate statistical power to achieve study objectives. Participants were included if they had a baseline score in the clinical range on the Generalized Anxiety Disorder 7-item scale (GAD-7; total score of ≥8) [25] or Patient Health Questionnaire 9-item scale (PHQ-9; total score ≥10) [26]. Individuals were excluded from the study if they did not have a baseline assessment that was completed ≤2 weeks before their first session and before their second session; or they did not complete at least 1 follow-up assessment either during care or a maximum of 5 weeks after the date of the final session. See the participant flow diagram in Figure 1 for more detail. Consistent with prior research, only clinical outcome assessments and sessions occurring within 1 SD of the average therapy episode duration (16.85 weeks) were included in this study.

### Clinical Program

#### Clinical Approach

A detailed description of LCT has been provided in prior research [18,21,22]. Briefly, the program consists of synchronous video therapy sessions combined with asynchronous guided practice sessions (ie, digital activities assigned by providers, therapist feedback, and direct client-provider messaging) via a secure, web-based digital care platform. The platform is accessible via web browser and mobile app. The program uses evidence-based clinical approaches with the highest-quality scientific evidence (eg, cognitive behavioral therapy, dialectical behavior therapy, and acceptance and commitment therapy) and is grounded in culturally responsive care principles to meet the social identity-related needs of a diverse client population [21,27-29].

#### Providers and Clinical Quality Supervision

Therapy is provided by licensed mental health professionals (eg, psychologists, marriage and family therapists, social workers, and professional counselors). Provider hiring is highly selective, emphasizing skillful use of evidence-based clinical practice and culturally responsive care. All providers receive >60 hours of intensive internal training upon hire, ongoing individual supervision, regular group consultation with a licensed clinical manager, and access to internal clinical consultation and training opportunities.

#### Guided Practice Sessions

**Figure 2:**
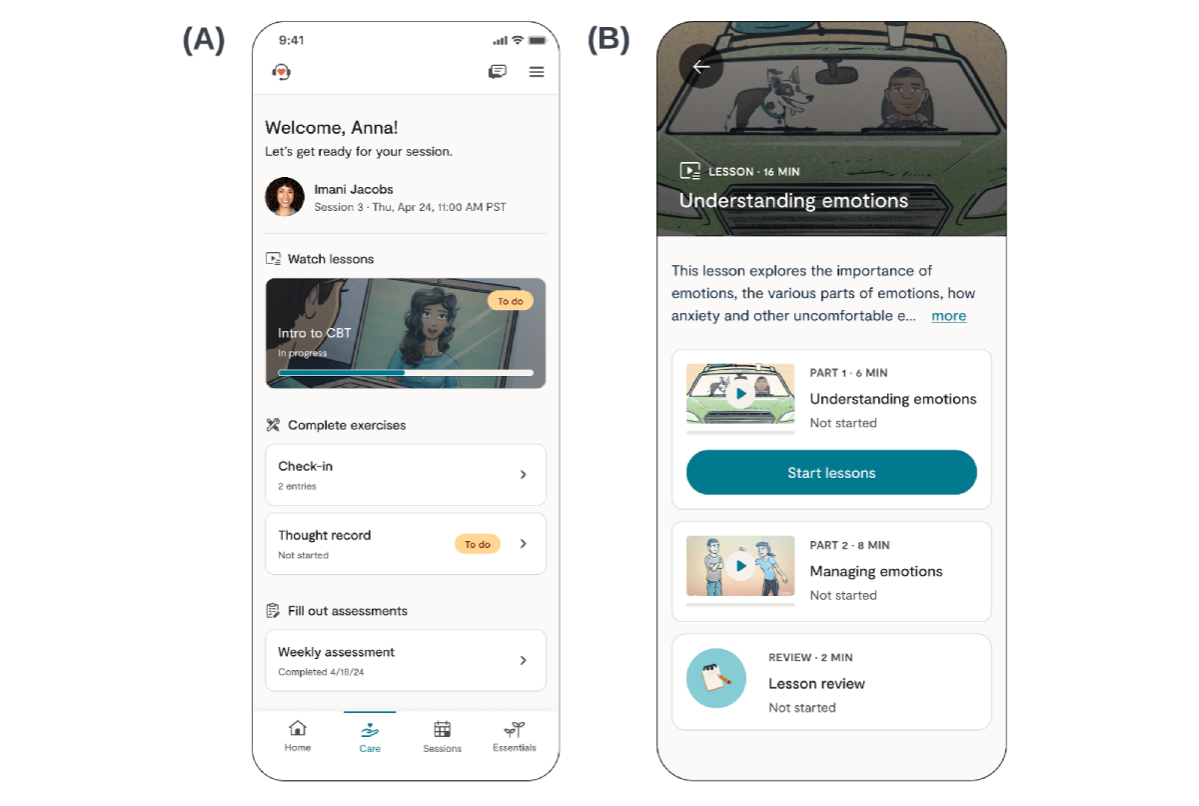
Illustrative examples of the client digital interface. (A) Client home page in mobile app (client name and provider’s name and image are fictional). (B) Client view of the “Understanding emotions” digital lesson.

Guided practice sessions refer to all interactions among the client, their provider, and the digital platform between synchronous sessions (see panels A and B in Figure 2 for illustrative examples of digital content). Two core elements of the guided practice sessions are digital video lessons and digital exercises. Lessons and exercises are based on evidence-based, transdiagnostic treatment approaches, including the Unified Protocol, dialectical behavior therapy, acceptance and commitment therapy, and other treatments rooted in cognitive behavioral principles [27-29]. Digital video lessons teach evidence-based therapeutic skills and concepts through a unique, narrative storytelling approach. Each lesson consists of 1-2 videos and concludes with a multiple-choice knowledge review to check clients’ comprehension. Digital exercises provide opportunities for clients to apply concepts and practice skills learned in therapy or in digital lessons and reflect on how these concepts apply to their daily lives. Providers have access to a large library of digital lessons and exercises for their clients, which apply to a range of presenting concerns. Providers collaborate with clients to select relevant and clinically appropriate digital activities for each guided practice session. Providers can also tailor activity instructions for each client. This process allows for personalization of guided practice sessions based on a client’s unique needs. Prior research has shown that use of these digital tools in LCT is associated with greater reductions in symptoms of anxiety and depression [22,30].

As clients complete their digital lessons and exercises, providers can view client progress and send asynchronous feedback messages. This provider feedback is intended to reinforce client learning and encourage consistent engagement for maximal clinical benefit [5,11]. The platform also allows for direct message exchanges between clients and providers. Through direct messages, clients can ask questions about their digital activities and ask their providers for help to troubleshoot challenges encountered when practicing skills. Providers can send check-in messages, answer questions, provide encouragement, and address any logistical or scheduling needs between sessions.

Finally, all clients receive validated clinical outcome assessments throughout care to monitor treatment progress. Both providers and clients are able to view clients’ responses, assessment scores, and trends over time, which supports measurement-based care.

### Measures

#### Demographic Characteristics, Session Attendance, and Treatment Duration

Clients may optionally self-report race, ethnicity, and gender identity during the intake process. Participant age was documented as age in years at the time of the first synchronous video session. Session attendance was automatically tracked on the platform. Treatment duration was computed as the time in weeks between the first session and the last session.

#### Digital Engagement During Guided Practice Sessions

Engagement with each element of guided practice sessions (client digital lesson completion, client digital exercise completion, provider feedback messages, and client-provider direct messages) was recorded automatically in the platform.

#### Clinical Outcome Measures

For quality assurance and clinical assessment to support measurement-based care, all clients in the program received the PHQ-9 [26] and GAD-7 [31] on a weekly basis and after the final session. For the purposes of this manuscript, client responses to these assessments are referred to as clinical outcome assessments. Reliable improvement was defined as a reduction ≥6 on the PHQ-9 and ≥4 on the GAD-7 [32,33]. Recovery was defined by a final score <10 for the PHQ-9 and <8 for the GAD-7 [25,26]. Reliable improvement and recovery were evaluated only for the measures on which the baseline score was in the clinical range for a given client. If a client scored in the clinical range on both the GAD-7 and the PHQ-9, they were included in analyses for both measures (including growth curve modeling). Growth curve models used all available clinical outcome assessments for each participant, including but not limited to the first and last measurements completed within the data collection period (see “Participants” section for more information).

### Statistical Analysis

All statistical analyses were performed in Python (version 3.10.9; Python Software Foundation) and R 4.2.3 (R Core Team) [34]. The statistical packages used for analyses are described in the remainder of the “Statistical Analysis” section where applicable.

#### Overall Treatment Outcome

Observed change in PHQ-9 and GAD-7 scores across the LCT episode was examined with 2-tailed dependent samples *t* tests, examining the difference between each individual’s baseline and last available clinical outcome assessments (a=.05). The SciPy package (version 1.10.0) was used for analysis [35].

#### Symptom Trajectories and Time-Varying Effects of Treatment Engagement

Symptom change throughout the LCT episode was evaluated using linear mixed-effects models to fit individual growth curves for the PHQ-9 and GAD-7, respectively, using the lme4 package (version 1.1.35) and full-information restricted maximum likelihood estimation [36]. Data explorations were conducted to evaluate whether data missingness patterns for the clinical outcome assessments of interest were consistent with the missing at random assumption and are summarized in Multimedia Appendix 2. Results were consistent with those expected if the missing at random assumption was met.

For growth curve models, model 1 included linear and quadratic fixed effects for the continuous time variable (weeks). Participant-level random effects were estimated for the intercept, linear, and quadratic time components. In model 2, binary indicator variables (ie, absent=0, present=1) were used to model the time-varying effects of engagement with each treatment component (therapy sessions, client digital lesson completion, client digital exercise completion, and provider feedback messages) during the past 7 days. Direct messages were not included as a time-varying covariate (TVC) effect in growth curve models, because not all direct messages were expected to have therapeutic content and therefore their effects on clinical outcomes would be heterogeneous. In model 3, additional TVCs were added for treatment engagement over the prior 8-14 days. This modeling approach allowed for testing of whether engagement with each program element was uniquely associated with greater symptom reduction in the subsequent 1-2 weeks.

Supplemental analyses were conducted using count variables for the treatment engagement TVCs, in place of the binary covariates used in the primary analysis. This would allow for testing of whether engagement with treatment components was associated with clinical outcomes in a linear manner, such that each additional engagement with the same treatment element in a given 1-2 weeks was associated with a fixed incremental benefit for symptom reduction. This approach is consistent with prior evaluations of this program but is limited in that it treats every digital element as an equal unit (ie, assumes a linear dose-response relationship). Findings from these analyses are reported in Multimedia Appendix 3.

An additional sensitivity analysis was conducted for the binary covariate models with participant demographics (age, gender identity, and race and ethnicity) added as fixed effects. Significant effects emerged for these covariates; however, addition of these variables provided only marginal improvement in model fit and did not change the pattern of results for the primary outcomes of interest. Results are reported in Multimedia Appendix 3.

## Results

### Participant Characteristics

A total of 33,492 clients were included in the sample (see Figure 1 for participant flow). Participants self-identified as Asian or Pacific Islander (5401/33,492, 16.1%), Black or African American (3026/33,492, 9.0%), Hispanic or Latino (3709/33,492, 11.1%), White (17,347/33,492, 51.8%), multiple identities (2687/33,492, 8.0%), or another identity (683/33,492, 2.0%). Participants self-reported their gender identities as female (21,217/33,492, 63.4%), male (10,547/33,492, 31.5%), nonbinary (295/33,492, 0.9%), and other identities (262/33,492, 0.8%). Nearly 9 in 10 participants (30,006/33,492, 89.5%) screened positive for anxiety on the GAD-7 [25] (score ≥8 at baseline), and nearly two-thirds (22,070/33,492, 65.9%) screened positive for depression on the PHQ-9 [26] (baseline score ≥10). See Table 1 for additional details on participant characteristics.

Participants who were excluded due to insufficient clinical outcome assessment data were compared with included participants on baseline demographics, baseline clinical severity, and session count. As expected, given the large sample size, statistically significant differences were found, but effect sizes were very small for demographic and clinical variables (Cohen *d*=0.06-0.07, Cramer *V*=.02-.05). Notably, the median session count for the excluded group was 1 (IQR 1-2), which is much smaller than that for included participants (median 6, IQR 4-8). This indicates that most excluded clients did not continue therapy after attending the intake session (see Multimedia Appendix 4 for full results).

**Table 1:** Participant characteristics.

			Entire sample (N=33,492)		Anxiety sample (baseline GAD-7^a^ ≥8) (N=30,006)	Depression sample (baseline PHQ-9^b^ ≥10) (N=22,070)
Age (years), mean (SD)			33.67 (9.51)		33.60 (9.44)	33.59 (9.65)
**Gender, n (%)**						
	Female	21,217 (63.35)		19,061 (63.52)		13,945 (63.19)
	Male	10,547 (31.49)		9385 (31.28)		6929 (31.40)
	Nonbinary	295 (0.88)		264 (0.88)		236 (1.07)
	Other	262 (0.78)		229 (0.76)		191 (0.87)
	Missing/unknown	1171 (3.50)		1067 (3.56)		769 (3.48)
**Race and ethnicity, n (%)**						
	Asian or Pacific Islander	5401 (16.13)		4867 (16.22)		3417 (15.48)
	Black or African American	3026 (9.03)		2704 (9.01)		2169 (9.83)
	Hispanic or Latino	3709 (11.07)		3335 (11.11)		2638 (11.95)
	Multiple	2687 (8.02)		2392 (7.97)		1837 (8.32)
	Other	683 (2.04)		633 (2.11)		452 (2.05)
	White	17,347 (51.79)		15,506 (51.68)		11,131 (50.43)
	Prefer not to disclose/missing	639 (1.91)		569 (1.90)		426 (1.93)
**Baseline GAD-7 severity, n (%)**						
	Minimal (0-4)	918 (2.74)		N/A^c^		918 (4.16)
	Mild/below clinical cutoff (5-7)^d^	2568 (7.67)		N/A^c^		2568 (11.64)
	Mild/above clinical cutoff (8-9)^d^	6296 (18.8)		6296 (20.98)		2531 (11.47)
	Moderate (10-14)	13,298 (39.71)		13,298 (44.32)		7684 (34.82)
	Severe (15-21)	10,412 (31.09)		10,412 (34.70)		8369 (37.92)
**Baseline PHQ-9 severity, n (%)**						
	Minimal (0-4)	2748 (8.20)		2748 (9.16)		N/A
	Mild (5-9)	8674 (25.90)		8674 (28.91)		N/A
	Moderate (10-14)	11,977 (35.76)		9430 (31.43)		11,977 (54.27)
	Moderately severe (15-19)	6996 (20.89)		6196 (20.65)		6996 (31.70)
	Severe (20-27)	3097 (9.25)		2958 (9.86)		3097 (14.03)
Baseline GAD-7, mean (SD)			12.28 (4.26)		13.08 (3.70)	12.67 (4.71)
Baseline PHQ-9, mean (SD)			11.85 (5.35)		11.71 (5.54)	14.80 (3.91)
Final GAD-7, mean (SD)			6.04 (4.46)		6.25 (4.51)	6.44 (4.69)
Final PHQ-9, mean (SD)			5.82 (4.93)		5.83 (4.98)	6.89 (5.21)
Treatment duration in weeks, median (IQR)			8.00 (4.00-12.71)		8.00 (4.00-12.57)	7.86 (3.86-12.43)

^a^GAD-7: Generalized Anxiety Disorder 7-item scale.

^b^PHQ-9: Patient Health Questionnaire 9-item scale.

^c^N/A: not applicable; no participants scored in this range within the participant subsample.

^d^To be included in the anxiety sample, clients were required to have a GAD-7 score above the clinical cutoff of 8 or higher. The mild severity category was therefore split to distinguish between those above and below the clinical cutoff.

### Treatment Characteristics and Overall Clinical Outcome

Participants attended a median of 6.0 live synchronous therapy sessions (IQR 4.0-8.0) over the course of a median of 8.0 (IQR 4.0-12.7) weeks. Participant engagement with digital tools during guided practice sessions was also high. Over the course of a therapy episode, participants viewed a median of 6.0 digital lessons (IQR 3.0-9.0), completed 6.0 exercises (IQR 2.0-12.0), and exchanged 16.0 direct messages (IQR 9.0-28.0) with their providers during guided practice sessions. In addition, participants received a median of 2.0 (IQR 0-4.0) feedback messages from their providers on their completed exercises. Descriptive statistics for engagement with all treatment components are detailed in Table 2.

Differences in symptom severity from the first to last clinical outcome assessment were evaluated via dependent samples *t* tests. Results indicated that participants experienced statistically significant reductions in symptoms of anxiety (n=30,006, t_30,005_=236.98; *P*<.001) and depression (n=22,070, t_22,069_=213.56; *P*<.001), as measured by the GAD-7 and PHQ-9, respectively, with very large observed effect sizes (Cohen *d*=1.37 for GAD-7; Cohen *d*=1.44 for PHQ-9). Furthermore, among the full sample, 86.6% (29,012/33,492) of participants experienced clinical improvement (defined as experiencing reliable improvement or recovery on the GAD-7, PHQ-9, or both) from their first to last assessment (Table 3).

**Table 2:** Session attendance and engagement with guided practice session elements^a^.

Treatment component			Full sample (N=33,492)	Anxiety sample (baseline GAD-7^b^ ≥8) (N=30,006)		Depression sample (baseline PHQ-9^c^ ≥10) (N=22,070)
**Synchronous therapy sessions**						
	Sessions attended	6.0 (4.0-8.0)		6.0 (4.0-8.0)	6.0 (4.0-8.0)	
**Guided practice sessions: provider engagement**						
	Lessons assigned	8.0 (6.0-11.0)		8.0 (6.0-11.0)	8.0 (6.0-12.0)	
	Exercises assigned	7.0 (4.0-12.0)		7.0 (4.0-12.0)	8.0 (4.0-12.0)	
	Exercise feedback messages sent	2.0 (0.0-4.0)		1.0 (0.0-4.0)	2.0 (0.0-4.0)	
	Direct messages sent	10.0 (6.0-16.0)		10.0 (6.0-16.0)	10.0 (6.0-16.0)	
**Guided practice sessions: client engagement**						
	Lessons completed	6.0 (3.0-9.0)		6.0 (3.0-9.0)	6.0 (3.0-9.0)	
	Exercises completed	6.0 (2.0-12.0)		6.0 (2.0-12.0)	6.0 (2.0-12.0)	
	Direct messages sent	6.0 (3.0-12.0)		6.0 (3.0-12.0)	6.0 (3.0-12.0)	
	Clinical outcome assessments completed (GAD-7 or PHQ-9)^d^	12.0 (8.0-18.0)		6.0 (4.0-9.0)	6.0 (4.0-9.0)	

^a^All descriptive statistics are reported as median (IQR) due to nonnormal distributions of variables within the sample. Engagement is totaled across a full episode of care.

^b^GAD-7: Generalized Anxiety Disorder 7-item scale.

^c^PHQ-9: Patient Health Questionnaire 9-item scale.

^d^For the full sample, each unique clinical outcome assessment completed was counted. GAD-7 and PHQ-9 measures were counted separately. For the anxiety and depression subsamples, only the relevant clinical outcome measures were included in assessment counts (ie, GAD-7 for anxiety subsample, PHQ-9 for depression subsample).

**Table 3:** Rates of reliable improvement and recovery^a^.

Baseline symptoms	n	Reliable improvement, n (%)	Recovery, n (%)	Reliable improvement and recovery, n (%)	Reliable improvement or recovery, n (%)
Anxiety symptoms (GAD-7^b^ ≥8)	30,006	22,807 (76.01)	20,748 (69.15)	18,955 (63.17)	24,600 (81.98)
Depression symptoms (PHQ-9^c^ ≥10)	22,070	15,122 (68.52)	16,208 (73.44)	13,644 (61.82)	17,686 (80.14)
Anxiety and depression symptoms (GAD-7 ≥8 and PHQ-9 ≥10)	18,584	11,411 (61.40)	10,845 (58.36)	13,036 (70.15)	16,304 (87.73)
Anxiety or depression symptoms (full sample)	33,492	26,672 (79.64)	26,111 (77.96)	23,255 (69.43)	29,012 (86.62)

^a^Reliable improvement was defined by meeting at least one of the following criteria: (1) ≥4-point decrease on the final GAD-7 among those with baseline GAD-7 score of ≥8 and (2) ≥6-point decrease on the final PHQ-9 among those with baseline PHQ-9 score of ≥10. Recovery was defined by meeting at least one of the following criteria: (1) final GAD-7 score of <8 among those with baseline GAD-7 score of ≥8 and (2) final PHQ-9 score of <10 among those with baseline score of ≥10.

^b^GAD-7: Generalized Anxiety Disorder 7-item scale.

^c^PHQ-9: Patient Health Questionnaire 9-item scale.

### Anxiety Symptom Trajectories and Time-Varying Effects of Engagement

Results from individual growth curve modeling for anxiety symptom trajectories and the time-varying effects of engagement with each program element are summarized in this section. Full modeling results are shown in Table 4. Individuals with initial GAD-7 scores in the clinical range were included in the analysis (n=30,006).

**Figure 3:**
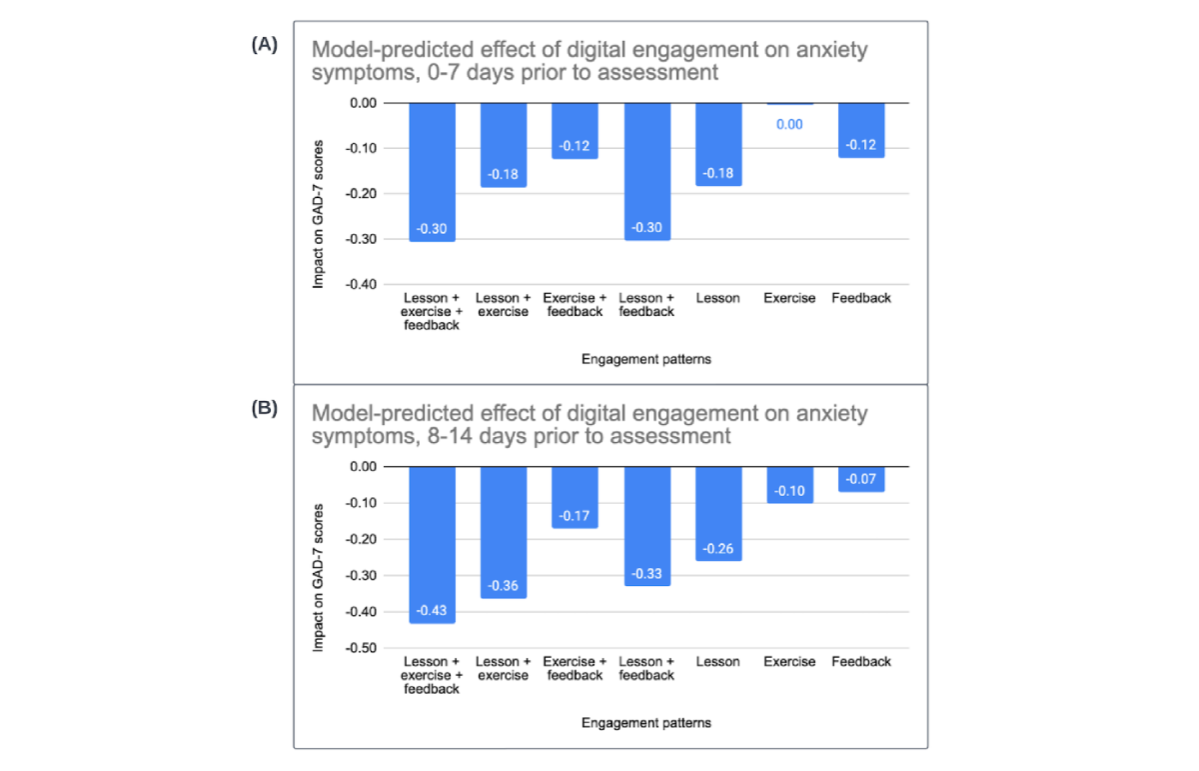
Estimated GAD-7 score reductions associated with different combinations of client and provider digital engagement in the (A) 0-7 days and (B) 8-14 days prior to a clinical outcome assessment. GAD-7: Generalized Anxiety Disorder-7 item scale.

Coefficients from an initial model examined linear and quadratic fixed effects of time in weeks on GAD-7 scores (model 1). On average, participants exhibited an initial decline in anxiety symptoms of more than 1 unit per week (*b*=–1.23, 95% CI –1.24 to –1.22) that attenuated over time (*b*=0.06, 95% CI 0.06-0.06). Model 2 incorporated fixed effects for each TVC indicating the presence (or absence) of a given program element during the 7 days prior to the clinical outcome assessment. The occurrence of 1 or more synchronous video sessions was significantly associated with a –0.70 unit decrease in anxiety scores (*b*=–0.70, 95% CI –0.73 to –0.67). The coefficients for TVCs identifying client completion of 1 or more digital lessons (*b*=–0.20, 95% CI –0.24 to –0.17), client completion of 1 or more digital exercises (*b*=–0.13, 95% CI –0.17 to –0.09), and 1 or more provider feedback messages (*b*=–0.21, 95% CI –0.26 to –0.17) also indicated that these forms of client and provider engagement were uniquely and significantly associated with lower GAD-7 scores. A similar pattern emerged in model 3, which incorporated TVC effects during the 8-14 days prior to completion of the clinical outcome assessment. As expected, synchronous video session occurrence (*b*=–0.58, 95% CI –0.61 to –0.54), client digital lesson completion (*b*=–0.26, 95% CI –0.30 to –0.22), client digital exercise completion (*b*=–0.10, 95% CI –0.14 to –0.06), and the presence of provider feedback messaging (*b*=–0.07, 95% CI –0.12 to –0.02) were each significantly associated with lower GAD-7 scores. The pattern of findings for the 7-day coefficients was virtually unchanged from model 2, with the exception of the coefficient for exercise completion in the 7 days prior to a clinical outcome assessment. In model 2, the coefficient was larger, negative, and statistically significant, whereas in model 3, it was not statistically different from 0. Likelihood ratio tests across these models (all *P* values of <.01), as well as information criteria indices (Akaike information criterion [AIC] and Bayesian information criterion [BIC]), suggested that model 3 provided the best fit to the observed data. Panels A and B in Figure 3 depict the possible combinations of digital engagement and their expected impact on clinical symptoms in the 2 weeks leading up to the clinical outcome assessment, based on the coefficients from model 3.

**Table 4:** Anxiety symptom trajectories and time-varying effects of engagement with elements of blended care therapy^a^.

	Model 1		Model 2			Model 3	
	*b* (95% CI)	*P* value	*b* (95% CI)	*P* value	*b* (95% CI)		*P* value
Intercept	11.50 (11.45 to 11.54)	<.001	11.92 (11.87 to 11.97)	<.001	12.10 (12.05 to 12.15)		<.001
Weeks	–1.23 (–1.24 to –1.22)	<.001	–1.17 (–1.18 to –1.15)	<.001	–1.05 (–1.06 to –1.03)		<.001
Weeks²	0.06 (0.06 to 0.06)	<.001	0.05 (0.05 to 0.05)	<.001	0.04 (0.04 to 0.05)		<.001
Therapy sessions last 7 days	N/A^b^	N/A	–0.70 (–0.73 to –0.67)	<.001	–0.82 (–0.85 to –0.78)		<.001
Digital lessons last 7 days	N/A	N/A	–0.20 (–0.24 to –0.17)	<.001	–0.18 (–0.22 to –0.15)		<.001
Digital exercises last 7 days	N/A	N/A	–0.13 (–0.17 to –0.09)	<.001	0.00 (–0.04 to 0.04)		.89
Provider feedback last 7 days	N/A	N/A	–0.21 (–0.26 to –0.17)	<.001	–0.12 (–0.16 to –0.08)		<.001
Therapy sessions 8-14 days	N/A	N/A	N/A	N/A	–0.58 (–0.61 to –0.54)		<.001
Digital lessons 8-14 days	N/A	N/A	N/A	N/A	–0.26 (–0.30 to –0.22)		<.001
Digital exercises 8-14 days	N/A	N/A	N/A	N/A	–0.10 (–0.14 to –0.06)		<.001
Provider feedback 8-14 days	N/A	N/A	N/A	N/A	–0.07 (–0.12 to –0.02)		.004
Deviance (–2LL^c^)	1,059,750	N/A	1,056,354	N/A	1,054,123		N/A
AIC^d^	1,059,772	N/A	1,056,384	N/A	1,054,161		N/A
BIC^e^	1,059,884	N/A	1,056,536	N/A	1,054,355		N/A

^a^The outcome of interest was client scores on the Generalized Anxiety Disorder 7-item scale (GAD-7). The analyzed sample included 30,006 individuals with a baseline GAD-7 score ≥8. *df*_residual_=196,023 for model 1, *df*_residual_=196,019 for model 2, and *df*_residual_=196,015 for model 3.

^b^Not applicable.

^c^–2LL: –2 × log likelihood.

^d^AIC: Akaike information criterion.

^e^BIC: Bayesian information criterion.

### Depression Symptom Trajectories and Time-Varying Effects of Engagement

Results from individual growth curve modeling for depression symptom trajectories and time-varying effects of engagement with each program element are summarized in this section. Full modeling results are reported in Table 5. Participants with initial PHQ-9 scores in the clinical range were included in the analysis (n=22,070).

**Figure 4:**
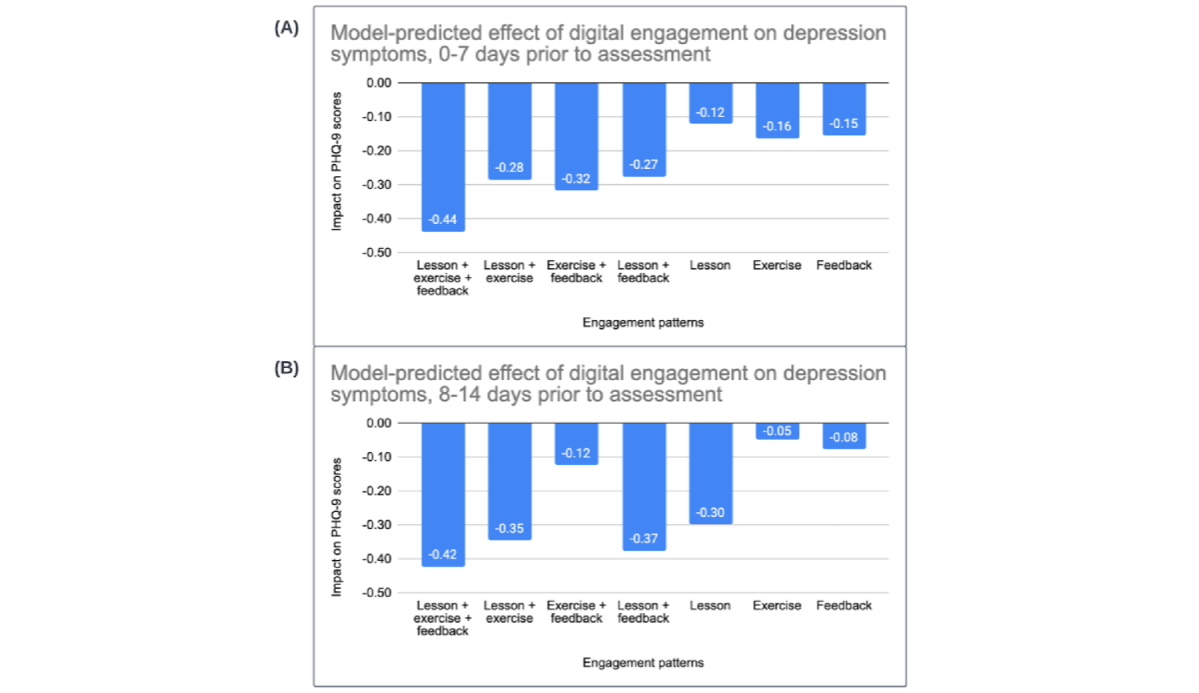
Estimated PHQ-9 score reductions associated with different combinations of client and provider digital engagement in the (A) 0-7 days and (B) 8-14 days prior to a clinical outcome assessment. PHQ-9: Patient Health Questionnaire—9 item scale.

Coefficients from an initial model examined linear and quadratic fixed effects of time in weeks on PHQ-9 scores (model 1). The coefficients in model 1 were consistent with a steep initial decline in depression symptoms of more than 1 unit per week (*b*=–1.45, 95% CI –1.46 to –1.43) that attenuated over time (*b*=0.07, 95% CI 0.07-0.07). The TVC effects in model 2 indicated that the occurrence of 1 or more synchronous video sessions during the previous week was associated with a –0.76 unit decrease in PHQ-9 scores (*b*=–0.76, 95% CI –0.80 to –0.72). Similarly, the past-week coefficients for client completion of 1 or more digital lessons (*b*=–0.14, 95% CI –0.19 to –0.10), client completion of 1 or more digital exercises (*b*=–0.31, 95% CI –0.36 to –0.26), and the occurrence of 1 or more provider feedback messages (*b*=–0.25, 95% CI –0.30 to –0.19) indicated that these forms of client and provider engagement were uniquely and significantly associated with lower PHQ-9 scores. In model 3, the coefficients for the occurrence of 1 or more synchronous video sessions (*b*=–0.67, 95% CI –0.71 to –0.62) 8-14 days prior to the clinical outcome assessment, as well as client completion of 1 or more digital lessons (*b*=–0.30, 95% CI –0.34 to –0.25), and the presence of feedback messaging from providers (*b*=–0.08, 95% CI –0.14 to –0.02), suggest that these forms of engagement are uniquely and significantly associated with lower PHQ-9 scores. In contrast, the coefficient for client digital exercise completion in the past 8-14 days was not significant (*b*=–0.05, 95% CI –0.10 to 0.01). The 7-day coefficients for therapy sessions, client lesson completion, and provider feedback messages were very similar across models 2 and 3. The coefficient for client exercise completion was numerically smaller relative to model 2 but still statistically significant (*b*=–0.15, 95% CI –0.21 to –0.10). Likelihood ratio tests across these models (all *P* values of <.01), as well as information criteria indices (AIC and BIC), suggested that model 3 provided the best fit to the observed data. Panels A and B in Figure 4 depict the possible combinations of digital engagement and their expected impact on clinical symptoms in the 2 weeks leading up to the clinical outcome assessment, based on the coefficients from model 3.

**Table 5:** Depression symptom trajectories and time-varying effects of engagement with elements of blended care therapy^a^.

	Model 1		Model 2			Model 3	
	*b* (95% CI)	*P* value	*b* (95% CI)	*P* value	*b* (95% CI)		*P* value
Intercept	12.90 (12.84 to 12.96)	<.001	13.39 (13.33 to 13.46)	<.001	13.59 (13.53 to 13.66)		<.001
Weeks	–1.45 (–1.46 to –1.43)	<.001	–1.36 (–1.38 to –1.34)	<.001	–1.23 (–1.25 to –1.21)		<.001
Weeks²	0.07 (0.07 to 0.07)	<.001	0.06 (0.06 to 0.06)	<.001	0.05 (0.05 to 0.05)		<.001
Therapy sessions last 7 days	N/A^b^	N/A	–0.76 (–0.80 to –0.72)	<.001	–0.89 (–0.93 to –0.85)		<.001
Digital lessons last 7 days	N/A	N/A	–0.14 (–0.19 to –0.10)	<.001	–0.12 (–0.16 to –0.08)		<.001
Digital exercises last 7 days	N/A	N/A	–0.31 (–0.36 to –0.26)	<.001	–0.16 (–0.21 to –0.11)		<.001
Provider feedback last 7 days	N/A	N/A	–0.25 (–0.30 to –0.19)	<.001	–0.15 (–0.21 to –0.10)		<.001
Therapy sessions 8-14 days	N/A	N/A	N/A	N/A	–0.67 (–0.71 to –0.62)		<.001
Digital lessons 8-14 days	N/A	N/A	N/A	N/A	–0.30 (–0.34 to –0.25)		<.001
Digital exercises 8-14 days	N/A	N/A	N/A	N/A	–0.05 (–0.10 to 0.01)		.09
Provider feedback 8-14 days	N/A	N/A	N/A	N/A	–0.08 (–0.14 to –0.02)		.01
Deviance (–2LL^c^)	808,568.9	N/A	805,755.1	N/A	804,048.6		N/A
AIC^d^	808,590.9	N/A	805,785.1	N/A	804,086.6		N/A
BIC^e^	808,699.6	N/A	805,933.3	N/A	804,274.3		N/A

^a^The outcome of interest was client scores on the Patient Health Questionnaire 9-item scale (PHQ-9). The analyzed sample included 22,070 individuals with a baseline PHQ-9 score of ≥10. *df*_residual_=144,505 for model 1, *df*_residual_=144,501 for model 2, and *df*_residual_=144,497 for model 3.

^b^Not applicable.

^c^–2LL: –2 × log likelihood.

^d^AIC: Akaike information criterion.

^e^BIC: Bayesian information criterion.

## Discussion

### Principal Findings

This study evaluated a unique blended care therapy program for anxiety and depression, consisting of synchronous video sessions and asynchronous guided practice sessions. Guided practice sessions are designed to provide more integrated and engaging opportunities for new client learning and skills practice outside of synchronous sessions. Providers encourage clients’ practice through asynchronous feedback and messaging. This study’s analyses evaluated the associations between guided practice session engagement and symptom reduction, with a novel focus on the time-varying effects of asynchronous provider feedback on client symptom outcomes.

Results supported the unique contributions of provider feedback messages to symptom reductions during care. Specifically, the presence of provider feedback messaging in the past 7 days and 8-14 days, respectively, was related to greater reductions in anxiety (*b*_7days_=–0.12, *b*_8-14days_=–0.07) and depression (*b*_7days_=–0.15, *b*_8-14days_=–0.08), even when accounting for effects of client-completed digital lessons, client-completed digital exercises, and synchronous video sessions. Results also provided further support for the clinical effects of client-level engagement with guided practice session elements (ie, viewing digital lessons and completing digital exercises), beyond the effects of synchronous video session attendance. More specifically, completing 1 or more digital lessons in the past 7 days and 8-14 days, respectively, was associated with significantly greater reductions in symptoms of anxiety (*b*_7days_=–0.18, *b*_8-14days_=–0.26) and depression (*b*_7days_=–0.12, *b*_8-14days_=–0.30). Completing an exercise in the past 7 days was associated with significantly lower depression symptoms (*b*_7days_=–0.16), and completing an exercise in the past 8-14 days was associated with significantly lower anxiety symptoms (*b*_8-14days_=–0.10). Finally, attending 1 or more synchronous sessions in the past 7 and 8-14 days, respectively, was also associated with significantly lower anxiety (*b*_7days_=–0.82, *b*_8-14 days_=–0.58) and depression (*b*_7days_=–0.89, *b*_8-14 days_=–0.67). If all types of guided practice session engagement were present over the course of 2 weeks, the model estimates suggest an expected total symptom reduction of 0.73 points on the GAD-7 and 0.86 points on the PHQ-9 during that time. Considering that the average baseline score on each measure was approximately 13 at baseline for the GAD-7 and 15 for the PHQ-9, the level of engagement observed in our sample would be expected to have a clinically meaningful impact over the course of care. Altogether, these findings indicate that each element of LCT provides distinct clinical benefit. In combination, these components can support more effective care overall.

It is interesting to note that digital exercise engagement was found to be slightly differentially associated with reductions in symptoms of anxiety versus depression. Specifically, engagement with digital exercises in the past 8-14 days was significantly associated with lower anxiety symptoms, but there was no significant relationship observed between exercise engagement in the past 7 days and anxiety outcomes. A converse pattern was observed for depression symptoms. Engagement with digital exercises in the past 7 days was significantly associated with lower depression symptoms, but no significant effect of engagement in the past 8-14 days was observed. Together, these results suggest that the impact of exercise engagement on depression symptoms may be more immediate, and the impact of exercises on anxiety symptoms may be slightly delayed. Clinically, this pattern is logical. Among other intervention strategies, evidence-based treatment for depression typically involves behavioral activation, which is intended to provide immediate positive reinforcement to clients and can support improved mood [4,37]. Thus, any exercises that may increase behavioral activation would be expected to have a more immediate effect on symptoms of depression. In contrast, evidence-based anxiety treatment typically involves reducing avoidance and gradually increasing exposure to previously feared or avoided situations [27]. When addressing anxiety symptoms, completion of exercises that involve increasing approach behaviors (and reducing avoidance) could initially correspond to consistent levels of distress before eventually leading to habituation and hence reduced symptoms [38]. Future research on the LCT program could explore how different types of digital exercises may differentially impact symptoms throughout care and how their effects may differ across presenting concerns.

This is the first known study to demonstrate that *both* provider and client digital engagement via asynchronous guided practice sessions contribute to symptom reduction, beyond the effects of synchronous video sessions. Moreover, each core element of the LCT model contributed uniquely and meaningfully to symptom reduction, including synchronous sessions and all guided practice session elements evaluated (digital lessons, digital exercises, and digital provider feedback messages). These effects also corresponded to strong overall clinical treatment outcomes. Approximately 87% (29,012/33,492) of clients achieved clinical improvement (ie, reliable improvement or recovery) over a median of 6 live synchronous sessions, compared with 22%-67% improvement rates over 8-15 sessions (or even longer) in traditional mental health care settings [19,39-41]. This particular model of blended care therapy therefore may support more efficient and effective care. In line with the LCT program’s commitment to data-informed clinical practice and continuous quality improvement, these findings will also be used in the LCT program to inform clinical practice by providing further evidence to encourage client and provider use of all guided practice session elements as a means of supporting greater improvements in therapy.

One possible interpretation of these findings is that this blended care model extends opportunities for clients to engage with and benefit from the “active elements” of psychotherapy, outside of their synchronous video sessions [5]. In traditional therapy models, synchronous sessions typically last for only 1 hour every 1-2 weeks. These sessions are the only setting in which provider teaching of “active elements” and client understanding of this information can occur, and thus opportunities are limited. In this blended care model, synchronous sessions occur at a similar cadence, but providers also facilitate new learning and skills practice for clients during guided practice sessions. They do this by first tailoring the selection of digital activities for each client and providing personalized instructions to help clients apply the skills to their unique situation. Providers then support client engagement between synchronous sessions through asynchronous feedback and messages. In this way, provider teaching and client understanding are extended well beyond the traditional bounds of the synchronous sessions.

Exploring the mechanisms for *why* provider feedback was associated with lower anxiety and depression symptoms is beyond the scope of this study. Some hypotheses can be made from prior research on the relationship between in-session provider homework-related behaviors and client symptom outcomes. Even when homework is assigned in traditional therapy settings, research has shown that providers do not consistently follow up on it in the next session [10,24]. Timely review and feedback from providers is critical because it provides clients with positive reinforcement and a sense of mastery for accomplishing the very hard work of behavior change [11]. In LCT, prompt provider feedback messages may give this reinforcement and signal to the client that they are supported when times are challenging. Thoughtful and personalized provider feedback messages may also remind the client of the link between the individual assignment completed and the client’s broader goals for therapy, potentially enhancing motivation to continue practicing and improving. Finally, provider feedback may prompt a client to revisit the exercise or give the client a new perspective on their experience. In turn, this could facilitate consolidation of learning and ultimately support longer-lasting behavior change. In these ways, well-designed digital platforms and tools—particularly those that support provider-client interactions between therapy sessions—may offer effective solutions for key barriers to homework engagement in psychotherapy [42]. Future research evaluating the LCT program could explore whether quantitative evidence supports these potential mechanisms, as well as how the content and depth of messaging affects future client engagement and outcomes.

Results demonstrated a high level of engagement with key guided practice session elements. In turn, this provider and client digital engagement was linked to better anxiety and depression outcomes. This clear link between engagement and clinical outcomes is often overlooked in digital mental health research [43,44]. Demonstrating correspondence between engagement and clinical outcomes is imperative when evaluating blended care therapy models and digital mental health intervention components, especially given that digital engagement is typically lower than intended or desired for these interventions [44,45]. By bolstering the therapeutic impact that occurs between synchronous therapy sessions, providers can serve clients more effectively and efficiently (ie, in fewer synchronous sessions but with stronger clinical outcomes). In turn, more clients can be served and benefit from high-quality mental health care.

### Strengths and Limitations

This study is strengthened by the inclusion of a very large participant sample of more than 30,000 individuals, a sample size that is uncommon in psychotherapy research. The design of the digital platform provided the opportunity for analysis of highly detailed data on client engagement and clinical symptom outcomes throughout care. To date, there are no other known studies with this level of detail on treatment engagement and clinical outcomes in such a large clinical sample. The sample was also diverse in terms of racial and ethnic identity. The study applied a rigorous statistical analysis approach, using objectively measured platform and session engagement data for both clients and providers. The results also represent clinical outcomes from real-world implementation of this program throughout the United States, and this enhances external validity and generalizability.

The study also has several limitations. Results from the growth curve modeling analyses provide information on time-varying associations among provider and client engagement variables and clinical outcomes; thus, they do not allow for causal conclusions. In addition, due to the high sensitivity of the data involved, this study did not evaluate the effects of client-provider direct messaging, aside from provider feedback messages that were focused specifically on clients’ digital exercise completion. It is possible that other direct message exchanges during guided practice sessions could also contribute to clinical outcomes. However, the direct messaging category is heterogeneous, because it includes clinically focused messages as well as those that are administrative in nature (eg, focused on scheduling or logistics). Effects of client-provider direct messaging may be studied in the future if the content of these messages could be further classified without identifying participants, such that analyses could differentiate between message types.

### Conclusions

In sum, this study yielded strong evidence that this blended care therapy program provided effective and efficient mental health care for clients at a large scale, and that provider feedback during guided practice sessions contributed to these clinical outcomes. This program leverages innovative digital tools to enhance therapeutic engagement for both providers and clients between synchronous sessions, providing substantial clinical benefit for clients.

## Appendix

Multimedia Appendix 1 STROBE (Strengthening the Reporting of Observational Studies in Epidemiology) checklist.

Multimedia Appendix 2 Missing data exploration.

Multimedia Appendix 3 Extended growth curve modeling results.

Multimedia Appendix 4 Excluded participant characteristics.

## Abbreviations

AIC: Akaike information criterion
BIC: Bayesian information criterion
GAD-7: Generalized Anxiety Disorder 7-item scale
HIPAA: Health Insurance Portability and Accountability Act
LCT: Lyra Care Therapy
PHQ-9: Patient Health Questionnaire 9-item scale
STROBE: Strengthening the Reporting of Observational Studies in Epidemiology
TVC: time-varying covariate

## Data Availability

The data sets generated and/or analyzed during this study are not publicly available due to the fact that the data are related to the delivery of health care and subject to HIPAA (Health Insurance Portability and Accountability Act of 1996).

As a business associate under HIPAA, Lyra is permitted to use patient information only as outlined in our Business Associate Agreements with our customers, which are covered entities. Many of our customer agreements do not permit us to disclose deidentified patient data; given this, we are unable to share the deidentified data due to contractual restrictions.
