# Supplementary material for: Clinical Effects of Asynchronous Provider-Guided Practice Sessions During Blended Care Therapy for Anxiety and Depression: Pragmatic Retrospective Cohort Study
Source: J Med Internet Res. 2024 Oct 18;26:e60502. doi: 10.2196/60502 (PMC11530739; doi:10.2196/60502)
Supplement: Multimedia Appendix 2 [file jmir_v26i1e60502_app2.docx]

**Summary of Explorations to Test whether Data are Consistent with Missing at Random Assumption**

To explore whether patterns of missingness in the data were supportive of the missing at random (MAR) assumption, this analysis examined whether observed variables included in the primary analysis models were predictive of missingness of the outcome variable during each week in care. To achieve this, the original data were transformed by aggregating outcomes into fixed intervals, namely, a weekly cadence. Specifically, for each individual participant, the episode was split into 1-week intervals, anchored around the initial session. Within each weekly interval, all clinical outcome measurements for either the GAD-7 or PHQ-9, respectively, were averaged to obtain a single score for the 1-week period. The window for week 16 was slightly larger than 1 week to extend to the end of the data inclusion period, up to 16.85 weeks.

According to Schafer and Graham [[43]](https://www.zotero.org/google-docs/?ccKsRF), the MAR assumption implies that the missingness of the outcome variable at week *j* is related only to other observed variables, including the outcome variable from all previous weeks (0 to *j*-1). This analysis therefore explored whether the pattern of missingness for the outcome variables (GAD-7 and PHQ-9 scores, respectively) at week *j* were predicted by other observed variables, including the outcomes observed at week *j*-1 and at week *j*-2, together with the demographic variables (age, gender, race and ethnicity), using logistic regression.

Results from this analysis are detailed in the summary table below. Briefly, the results suggested that the scores from all weeks were significantly predicted by other observed variables included in the growth curve models, for both the GAD-7 and the PHQ-9 samples, respectively. Although MAR can never be definitively proven, these analyses provide evidence that the patterns of missingness in our sample were consistent with the assumption of MAR, supporting the validity of the missing data handling procedures implemented by the mixed effects models used in the present study.

**Results: Predictors of Missingness by Week in Care**

Table 1. Predictors of missingness by week in care for data included in anxiety and depression growth curve models, respectively

|  | Anxiety Sample  (baseline GAD-7 ≥8; n=30,006) | | | Depression Sample  (baseline PHQ-9 ≥10; n=22,070) | | |
| --- | --- | --- | --- | --- | --- | --- |
| Week | Significant demographic predictors | Predicted by lag 1 observed scoresᵃ | Predicted by lag 2 observed scoresᵃ | Significant demographic predictors | Predicted by lag 1 observed scoresᵃ | Predicted by lag 2 observed scoresᵃ |
| 1 | Race and Ethnicity | Yes | - | No | Yes | - |
| 2 | Age, Gender, Race and Ethnicity | Yes | No | Age, Race and Ethnicity | Yes | No |
| 3 | Race and Ethnicity | Yes | No | Race and Ethnicity | Yes | No |
| 4 | No | Yes | No | No | Yes | Yes |
| 5 | Race and Ethnicity | Yes | No | Race and Ethnicity | Yes | Yes |
| 6 | No | Yes | No | No | Yes | No |
| 7 | Gender | Yes | No | Gender, Race and Ethnicity | Yes | No |
| 8 | Age | Yes | No | Race and Ethnicity | Yes | Yes |
| 9 | Gender | Yes | No | No | Yes | No |
| 10 | Race and Ethnicity | Yes | No | Age | Yes | No |
| 11 | Age | Yes | No | Age | Yes | No |
| 12 | Age | Yes | No | Age | No | No |
| 13 | Age | Yes | Yes | Age | Yes | No |
| 14 | No | Yes | No | Age | Yes | Yes |
| 15 | No | Yes | No | Gender | No | No |
| 16 | No | Yes | No | No | Yes | Yes |

^a^These columns summarize whether a statistically significant effect was detected for the 1-week and 2-week lagged values of GAD-7 and PHQ-9 scores, respectively, predicting missingness of the week *n* score (week noted in the leftmost column of the table). Predictors were determined to be statistically significant if *p*<.05.

## *Notes.* GAD-7 = Generalized Anxiety Disorder-7 item scale. PHQ-9 = Patient Health Questionnaire-9 item.
