# Supplementary material for: Clinical Effects of Asynchronous Provider-Guided Practice Sessions During Blended Care Therapy for Anxiety and Depression: Pragmatic Retrospective Cohort Study
Source: J Med Internet Res. 2024 Oct 18;26:e60502. doi: 10.2196/60502 (PMC11530739; doi:10.2196/60502)
Supplement: Multimedia Appendix 3 [file jmir_v26i1e60502_app3.docx]

## Supplemental Results: Growth Curve Models with Count-Based Time-Varying Covariates

Coefficients for the GAD-7 and PHQ-9 analyses using count variables for time-varying covariates (TVCs) are provided in the Supplemental Tables below in this Multimedia Appendix. A narrative summary of the findings are described below, along with a discussion of any differences in the pattern of results observed between the models with binary TVCs (primary results, Tables 4-5) and those with count-based TVCs.

#### Anxiety Symptoms

Coefficients from Model 1 indicated that on average, clients exhibited an initial decline in symptoms of more than 1 unit per week (b=-1.23, 95% CI -1.24, -1.22) that became flatter (less negative) over time (b=0.06, 95% CI 0.06, 0.06). Model 2 incorporated fixed effects for each TVC indicating the number of times an engagement element occurred during the 7 days prior to the clinical outcome assessment. The number of synchronous video sessions was associated with a -0.69 unit decrease in anxiety scores (b=-0.69, 95% CI -0.72, -0.66). The coefficients for TVCs identifying the number of client digital lessons completed (b=-0.12, 95% CI -0.14, -0.11) and provider feedback messages sent (b=-0.12, 95% CI -0.14, -0.10) also indicated that these forms of client and provider engagement were uniquely associated with lower GAD-7 scores. A small but significant positive coefficient for client digital exercises completed was observed (b=0.02, 95% CI 0.01, 0.04). A similar pattern emerged in Model 3, which incorporated TVC effects during the 8-14 days prior to the completion of the clinical outcome assessment. Specifically, the numbers of synchronous video sessions (b=-0.58, 95% CI -0.61, -0.54), client digital lessons completed (b=-0.15, 95% CI -0.17, -0.13), and provider feedback messages sent (b=-0.04, 95% CI -0.06, -0.02) were associated with lower GAD-7 scores, though the number of client exercises completed was not (b=-0.01, 95% CI -0.02, 0.01). The pattern of findings for the 7-day coefficients were very similar to Model 2. Likelihood ratio tests across these models (all *P*s<.01), as well as information criteria indices (AIC, BIC), suggested that Model 3 provided the best fit to the observed data.

For the GAD-7 analysis using count-based TVCs, the estimated fixed trajectory coefficients were nearly identical to the binary TVC analysis, however, the coefficients for the number of digital lessons completed by the client and the number of feedback messages from the provider during the 7 days prior to clinical outcome assessment were notably smaller relative to those reported in Model 2 of the primary analysis (though still statistically significant). In addition, each digital exercise completed by clients was associated with a very small but statistically significant increase in GAD-7 scores. In Model 3, the coefficients for client lessons completed and provider feedback messages sent were smaller than in the binary model, though they were still negative and statistically significant. The coefficient for client exercises completed was smaller relative to the binary model and was not statistically significant. In contrast, coefficients for the count TVC version of therapy sessions were not notably different from the binary versions presented in the primary results. Smaller coefficients are unsurprising in the count models relative to the binary models, given that the former models represent the effects of each individual unit of engagement with a specific program element (e.g., each individual digital lesson viewed) versus the combined effect of any and all engagement with that element occurring in a given timeframe.

#### Depression Symptoms

The coefficients in Model 1 were also consistent with a steep initial decline in depression symptoms of more than 1 unit per week (b=-1.45, 95% CI -1.46, -1.43) that became flatter (less negative) over time (b=0.07, 95% CI 0.07, 0.07). The TVC effects in Model 2 indicated that each synchronous video session during the previous week was associated with a -0.76 unit decrease in PHQ-9 scores (b=-0.76, 95% CI -0.80, -0.72), and that the completion of each digital lesson (b=-0.11, 95% CI -0.13, -0.09) or exercise (b=-0.02, 95% CI -0.04, -0.01) by clients, as well as the sending of each feedback message by the provider (b=-0.12, 95% CI -0.15, -0.10) were also associated with lower depression symptoms. In Model 3, fixed TVCs were added to evaluate the effects of engagement in the 8-14 days prior to clinical outcome assessment. The coefficients for the number of synchronous video sessions (b=-0.67, 95% CI -0.71, -0.63), the number of client-completed lessons (b=-0.15, 95% CI -0.17, -0.12), and the number of feedback messages from providers (b=-0.06, 95% CI -0.09, -0.03) suggest that these forms of engagement are uniquely associated with lower depression scores. In contrast, the coefficient for client digital exercises completed was not statistically significant (b=0.01, 95% CI -0.01, 0.03). The 7-day coefficients for therapy sessions, client lessons completed, and provider feedback messages sent were very similar across Models 2 and 3. The Model 3 coefficient for client exercises completed was positive and statistically significant (b=0.03, 95% CI 0.01, 0.04) but very small in magnitude. Likelihood ratio tests across these models (all *P*s<.01), as well as information criteria indices (AIC, BIC), suggested that Model 3 provided the best fit to the observed data.

Similar to the results for the GAD-7 analyses, the trajectory coefficients for the PHQ-9 analysis with count-based TVCs were identical to those of the binary TVC model. The coefficient for therapy sessions was virtually unchanged. In Model 2, the coefficient for client digital lesson completion was slightly smaller, whereas the coefficients for client exercises completed and provider feedback messaging were considerably weaker than in the binary versions. In Model 3, the coefficient for therapy sessions was identical to the binary TVC analysis. The observed effects for client digital lessons and provider feedback messages were notably smaller for the count TVC models (though still statistically significant). As stated above, smaller coefficients are to be expected for the count models relative to the binary models, given that the former models represent the effects of each individual unit within a specific treatment component (e.g., each individual digital lesson viewed) versus the combined effect of any and all aspects of that component occurring in a given timeframe.

### Supplemental Growth Curve Modeling Results Tables

Table 1. Supplemental anxiety trajectory modeling results (engagement covariates treated as count variables)

|  | Model 1 | | Model 2 | | Model 3 | |
| --- | --- | --- | --- | --- | --- | --- |
|  | *b* (95% CI) | *P* | *b* (95% CI) | *P* | *b* (95% CI) | *P* |
| Intercept | 11.50 (11.45 to 11.54) | <.001 | 11.88 (11.83 to 11.93) | <.001 | 12.08 (12.03 to 12.13) | <.001 |
| Week | -1.23 (-1.24 to -1.22) | <.001 | -1.18 (-1.20 to -1.17) | <.001 | -1.07 (-1.08 to -1.06) | <.001 |
| Week Squared | 0.06 (0.06 to 0.06) | <.001 | 0.06 (0.05 to 0.06) | <.001 | 0.05 (0.05 to 0.05) | <.001 |
| Therapy sessions last 7 days | - | - | -0.69 (-0.72 to -0.66) | <.001 | -0.81 (-0.84 to -0.78) | <.001 |
| Patients submitted digital lessons last 7 days | - | - | -0.12 (-0.14 to -0.11) | <.001 | -0.13 (-0.14 to -0.11) | <.001 |
| Patients submitted digital exercises last 7 days | - | - | 0.02 (0.01 to 0.04) | <.001 | 0.07 (0.06 to 0.08) | <.001 |
| Providers feedback last 7 days | - | - | -0.12 (-0.14 to -0.10) | <.001 | -0.09 (-0.11 to -0.06) | <.001 |
| Therapy sessions 8-14 days | - | - | - | - | -0.58 (-0.61 to -0.54) | <.001 |
| Patients submitted digital lessons 8-14 days | - | - | - | - | -0.15 (-0.17 to -0.13) | <.001 |
| Patients submitted digital exercises 8-14 days | - | - | - | - | -0.01 (-0.02 to 0.01) | .47 |
| Providers feedback 8-14 days | - | - | - | - | -0.04 (-0.06 to -0.02) | .001 |
| Deviance (-2LL) | 1059750 | - | 1056467 | - | 1054016 | - |
| AIC | 1059772 | - | 1056497 | - | 1054054 | - |
| BIC | 1059884 | - | 1056650 | - | 1054248 | - |

*Notes.* The clinical outcome of interest was client scores on the Generalized Anxiety Disorder-7 item scale (GAD-7). The analyzed sample included *n*=30,006 individuals with a baseline GAD-7 score ≥8.

Table 2. Supplemental depression trajectory modeling results (engagement covariates treated as count variables)

|  | Model 1 | | Model 2 | | Model 3 | |
| --- | --- | --- | --- | --- | --- | --- |
|  | *b* (95% CI) | *P* | *b* (95% CI) | *P* | *b* (95% CI) | *P* |
| Intercept | 12.90 (12.84 to 12.96) | <.001 | 13.34 (13.28 to 13.40) | <.001 | 13.56 (13.50 to 13.63) | <.001 |
| Week | -1.45 (-1.46 to -1.43) | <.001 | -1.38 (-1.40 to -1.37) | <.001 | -1.26 (-1.27 to -1.24) | <.001 |
| Week^2^ | 0.07 (0.07 to 0.07) | <.001 | 0.06 (0.06 to 0.07) | <.001 | 0.05 (0.05 to 0.06) | <.001 |
| Therapy sessions last 7 days | - | - | -0.76 (-0.80 to -0.72) | <.001 | -0.91 (-0.95 to -0.86) | <.001 |
| Patients submitted digital lessons last 7 days | - | - | -0.11 (-0.13 to -0.09) | <.001 | -0.10 (-0.12 to -0.08) | <.001 |
| Patients submitted digital exercises last 7 days | - | - | -0.02 (-0.04 to -0.01) | .002 | 0.03 (0.01 to 0.04) | <.001 |
| Providers feedback last 7 days | - | - | -0.12 (-0.15 to -0.10) | <.001 | -0.10 (-0.12 to -0.07) | <.001 |
| Therapy sessions 8-14 days | - | - | - | - | -0.67 (-0.71 to -0.63) | <.001 |
| Patients submitted digital lessons 8-14 days | - | - | - | - | -0.15 (-0.17 to -0.12) | <.001 |
| Patients submitted digital exercises 8-14 days | - | - | - | - | 0.01 (-0.01 to 0.03) | .27 |
| Providers feedback 8-14 days | - | - | - | - | -0.06 (-0.09 to -0.03) | <.001 |
| Deviance (-2LL) | 808568.9 | - | 805969.4 | - | 804095.8 | - |
| AIC | 808590.9 | - | 805999.4 | - | 804133.8 | - |
| BIC | 808699.6 | - | 806147.6 | - | 804321.5 | - |

*Notes.* The clinical outcome of interest was client scores on the Patient Health Questionnaire-9 item scale (PHQ-9). The analyzed sample included *n*=22,070 individuals with a baseline PHQ-9 score ≥10.

Table 3. Supplemental modeling results with demographic variables as covariates

|  | | Anxiety sample  (baseline GAD-7 ≥8) | | Depression sample  (baseline PHQ-9 ≥10) | |
| --- | --- | --- | --- | --- | --- |
|  | | *b* (95% CI) | *P* | *b* (95% CI) | *P* |
| Intercept | | 12.14 (12.08 to 12.21) | <.001 | 13.58 (13.49 to 13.67) | <.001 |
| Week | | -1.05 (-1.06 to -1.03) | <.001 | -1.23 (-1.25 to -1.21) | <.001 |
| Week² | | 0.04 (0.04 to 0.05) | <.001 | 0.05 (0.05 to 0.05) | <.001 |
| Therapy sessions last 7 days | | -0.82 (-0.85 to -0.78) | <.001 | -0.89 (-0.93 to -0.85) | <.001 |
| Digital lessons last 7 days | | -0.18 (-0.22 to -0.15) | <.001 | -0.12 (-0.16 to -0.08) | <.001 |
| Digital exercises last 7 days | | -0.00 (-0.04 to 0.03) | .84 | -0.16 (-0.21 to -0.12) | <.001 |
| Provider feedback last 7 days | | -0.12 (-0.16 to -0.08) | <.001 | -0.15 (-0.21 to -0.10) | <.001 |
| Therapy sessions 8-14 days | | -0.58 (-0.61 to -0.54) | <.001 | -0.67 (-0.71 to -0.62) | <.001 |
| Digital lessons 8-14 days | | -0.26 (-0.30 to -0.22) | <.001 | -0.30 (-0.34 to -0.25) | <.001 |
| Digital exercises 8-14 days | | -0.10 (-0.15 to -0.06) | <.001 | -0.05 (-0.10 to 0.01) | 0.09 |
| Provider feedback 8-14 days | | -0.07 (-0.12 to -0.02) | .004 | -0.08 (-0.14 to -0.02) | .01 |
| Age (mean-centered) | | -0.01 (-0.01 to -0.00) | <.001 | -0.01 (-0.02 to -0.01) | <.001 |
| **Gender (reference category: Female)** | |  |  |  |  |
|  | Male | -0.21 (-0.29 to -0.13) | <.001 | -0.23 (-0.33 to -0.13) | <.001 |
|  | Other | 0.41 (0.12 to 0.69) | .005 | 1.16 (0.81 to 1.51) | <.001 |
|  | Unknown/missing | 0.18 (-0.01 to 0.38) | .06 | 0.20 (-0.07 to 0.46) | .14 |
| **Race & ethnicity (reference category: White)** | |  |  |  |  |
|  | Asian or Pacific Islander | -0.13 (-0.24 to -0.03) | .01 | -0.07 (-0.22 to 0.07) | .30 |
|  | Black or African American | -0.05 (-0.19 to 0.08) | .42 | 0.03 (-0.14 to 0.20) | .70 |
|  | Hispanic or Latino | 0.06 (-0.06 to 0.18) | .30 | 0.22 (0.06 to 0.37) | .006 |
|  | Multiple | 0.20 (0.06 to 0.33) | .005 | 0.26 (0.09 to 0.44) | .004 |
|  | Other | 0.13 (-0.12 to 0.38) | .31 | 0.18 (-0.16 to 0.52) | .29 |
|  | Missing | 0.44 (0.18 to 0.70) | .001 | 0.59 (0.24 to 0.94) | <.001 |
| Deviance (-2LL) | | 1054030 | - | 803919.8 | - |
| AIC | | 1054088 | - | 803977.8 | - |
| BIC | | 1054383 | - | 804264.4 | - |

*Notes.* Clinical outcomes of interest were the Generalized Anxiety Disorder-7 item scale (GAD-7) and Patient Health Questionnaire-9 item scale (PHQ-9). Time-varying covariate effects were treated as binary effects. The analyzed GAD-7 sample included *n*=30,006 individuals with a baseline GAD-7 score ≥8. The analyzed PHQ-9 sample included *n*=22,070 individuals with a baseline PHQ-9 score ≥10.
