# Supplementary material for: Clinical Effects of Asynchronous Provider-Guided Practice Sessions During Blended Care Therapy for Anxiety and Depression: Pragmatic Retrospective Cohort Study
Source: J Med Internet Res. 2024 Oct 18;26:e60502. doi: 10.2196/60502 (PMC11530739; doi:10.2196/60502)
Supplement: Multimedia Appendix 4 [file jmir_v26i1e60502_app4.docx]

## Comparison of Included vs Excluded Participants on Baseline Characteristics and Session Count

The below table summarizes results from comparisons of baseline demographic characteristics (age, gender, race and ethnicity), baseline clinical severity (scores on the GAD-7 and PHQ-9, respectively), and session count for the full treatment episode.

As detailed in the table below, all group comparisons were found to be statistically significant, such that differences between included and excluded participants were detected. However, effect sizes for all demographic variables and clinical severity comparisons were negligible to small, suggesting that these differences were not clinically meaningful and most likely attributable to large sample size and a statistical power to detect very small differences. The exception to this pattern was the result for session count, where very large differences between groups were detected. When examining median session counts between the two groups, it appears that most excluded participants only attended one or two sessions and likely opted not to complete a full course of care.

Table 1. Results from comparisons of included vs excluded participants on demographic and clinical characteristics.

|  |  | Included Participants | Excluded Participants | Results for Statistical Comparisons of Included/Excluded Participants | | |
| --- | --- | --- | --- | --- | --- | --- |
|  |  | n=33492 | n=3510 | Test Statistic | *P* | Effect Size |
| Age, mean (SD) | | 33.67 (9.51) | 33.10 (9.04) | *t*₄₃₆₃=3.55 | <.001 | Cohen *d*=0.06 |
| **Gender, n (%)** | |  |  | *χ²*=11.86 | 0.018 | Cramer *V*=0.02 |
|  | Female | 21217 (63.35) | 2224 (63.36) |  |  |  |
|  | Male | 10547 (31.49) | 1064 (30.31) |  |  |  |
|  | Non-binary | 295 (0.88) | 35 (1.00) |  |  |  |
|  | Other | 262 (0.78) | 27 (0.77) |  |  |  |
|  | Missing/unknown | 1171 (3.50) | 160 (4.56) |  |  |  |
| **Race and Ethnicity, n (%)** | |  |  | *χ²*=92.44 | <.001 | Cramer *V*=0.05 |
|  | Asian or Pacific Islander | 5401 (16.13) | 703 (20.03) |  |  |  |
|  | Black or African American | 3026 (9.03) | 382 (10.88) |  |  |  |
|  | Hispanic or Latino | 3709 (11.07) | 366 (10.43) |  |  |  |
|  | Multiple | 2687 (8.02) | 257 (7.32) |  |  |  |
|  | Other | 683 (2.04) | 103 (2.93) |  |  |  |
|  | White | 17347 (51.79) | 1601 (45.61) |  |  |  |
|  | Prefer not to disclose / missing | 639 (1.91) | 98 (2.79) |  |  |  |
| Baseline GAD-7, mean (SD)ᵃ | | 12.28 (4.26) | 12.57 (4.32) | *t*₃₇₀₆=-3.65 | <.001 | Cohen *d*=-0.07 |
| Baseline PHQ-9, mean (SD)ᵃ | | 11.85 (5.35) | 12.22 (5.71) | *t*₃₆₄₅=-3.43 | 0.001 | Cohen *d*=-0.07 |
| Session Count, median (IQR) | | 6.00 (4.00-8.00) | 1.00 (1.00-2.00) | *U*=108949930.0 | <.001 | Rank Biserial *r*=-0.85 |

^a^Baseline symptom severity scores were available for n=3120 excluded patients with a valid baseline clinical outcome assessment.
